# Tetra­phenyl piperazine-1,4-diyldiphos­pho­nate

**DOI:** 10.1107/S1600536810047860

**Published:** 2010-11-24

**Authors:** Mehrdad Pourayoubi, Poorya Zargaran

**Affiliations:** a Department of Chemistry, Ferdowsi University of Mashhad, Mashhad, 91779, Iran

## Abstract

The mol­ecule of the title compound, C_28_H_28_N_2_O_6_P_2_, is organized around an inversion center located at the centre of the piperazine ring. Both piperazine N atoms are substituted by P(O)(OC_6_H_5_)_2_ phospho­ester groups. The P atoms display a slightly distorted tetra­hedral environment; the N atoms show some deviation from planarity. The O atoms of the P=O groups are involved in inter­molecular C—H⋯O hydrogen bonds, building *R*_2_^2^(22) rings, in extended chains parallel to the *a* axis. C—H⋯π inter­actions involving the phenyl rings further stabilize the packing.

## Related literature

For the physical properties of bis­phospho­ramidates, see: Nguyen & Kim (2008 ▶). For related structures, see: Chen *et al.* (2007 ▶); Balakrishna *et al.* (2003 ▶, 2006 ▶); Rodriguez i Zubiri *et al.* (2002 ▶). For hydrogen-bond motifs, see: Etter *et al.* (1990 ▶); Bernstein *et al.* (1995 ▶).
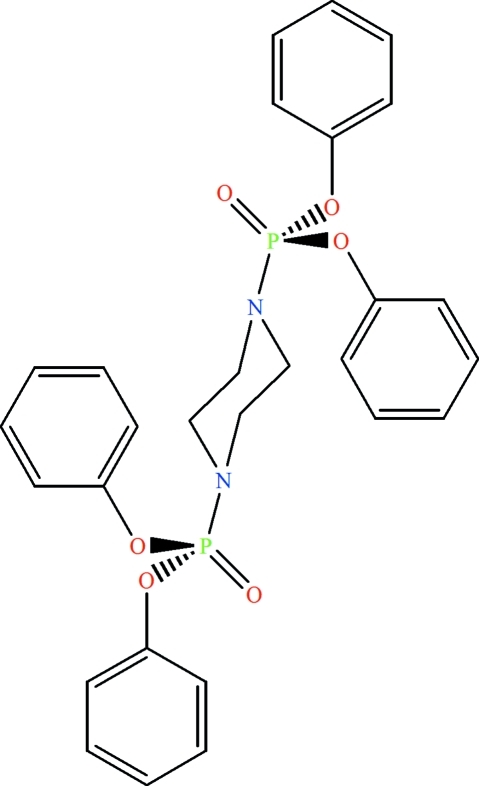


## Experimental

### 

#### Crystal data

C_28_H_28_N_2_O_6_P_2_
*M* *_r_* = 550.46
Monoclinic, 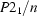

*a* = 6.3117 (4) Å
*b* = 8.9530 (5) Å
*c* = 22.8630 (13) Å
β = 96.756 (1)°
*V* = 1282.99 (13) Å^3^
*Z* = 2
Mo *K*α radiation
μ = 0.22 mm^−1^
*T* = 100 K
0.55 × 0.40 × 0.20 mm

#### Data collection

Bruker APEXII CCD area-detector diffractometer
Absorption correction: multi-scan (*SADABS*; Bruker, 2005 ▶) *T*_min_ = 0.890, *T*_max_ = 0.958
14864 measured reflections
3405 independent reflections
3071 reflections with *I* > 2σ(*I*)
*R*_int_ = 0.025

#### Refinement

*R*[*F* ^2^ > 2σ(*F* ^2^)] = 0.032
*wR*(*F* ^2^) = 0.089
*S* = 1.03
3405 reflections
172 parameters
H-atom parameters constrained
Δρ_max_ = 0.51 e Å^−3^
Δρ_min_ = −0.27 e Å^−3^

### 

Data collection: *APEX2* (Bruker, 2005 ▶); cell refinement: *SAINT* (Bruker, 2005 ▶); data reduction: *SAINT*; program(s) used to solve structure: *SHELXS97* (Sheldrick, 2008 ▶); program(s) used to refine structure: *SHELXL97* (Sheldrick, 2008 ▶); molecular graphics: *ORTEPIII* (Burnett & Johnson, 1996 ▶), *ORTEP-3 for Windows* (Farrugia, 1997 ▶) and *PLATON* (Spek, 2009 ▶); software used to prepare material for publication: *SHELXL97*.

## Supplementary Material

Crystal structure: contains datablocks I, global. DOI: 10.1107/S1600536810047860/dn2623sup1.cif

Structure factors: contains datablocks I. DOI: 10.1107/S1600536810047860/dn2623Isup2.hkl

Additional supplementary materials:  crystallographic information; 3D view; checkCIF report

## Figures and Tables

**Table 1:** Hydrogen-bond geometry (Å, °) *Cg*1 and *Cg*2 are the centroids of the C3–C8 and C11–C14 rings, respectively.

*D*—H⋯*A*	*D*—H	H⋯*A*	*D*⋯*A*	*D*—H⋯*A*
C14—H14*A*⋯O1^i^	0.95	2.49	3.4327 (15)	172
C11—H11*A*⋯*Cg*1^ii^	0.95	2.74	3.3324 (12)	121
C7—H7*A*⋯*Cg*2^iii^	0.95	2.59	3.4099 (13)	145

Symmetry codes: (i) 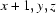; (ii) 
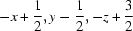
; (iii) 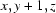.

**Fig. 1.**
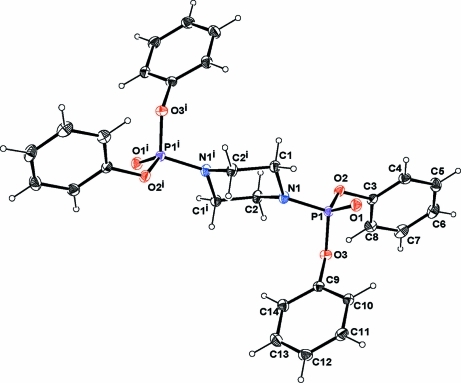
Molecular view with the atom labeling scheme. Displacement ellipsoids are drawn at the 50% probability level. H atoms are represented as small spheres of arbitrary radii.

**Fig. 2.**
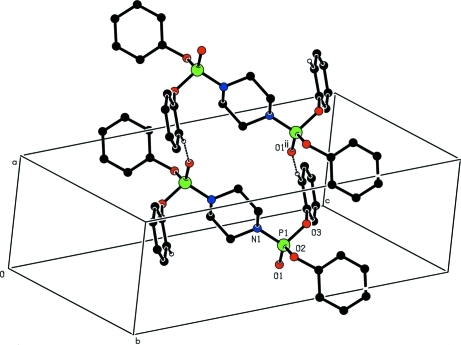
Partial packing view showing the formation of dimer through C–H···O interactions. H bonds are shown as dashed lines. [Symmetry codes: (ii) x+1, y, z]

## supplementary crystallographic information

### Comment

The thermal behavior and flame retardancies of some bisphosphoramidates, with a P(O)*X*P(O) skeleton, such as the title compound have been investigated by Nguyen & Kim (2008). Here, we report the crystal structure of the title compound.

The molecule of the title compound is organized around inversion center located in the middle of the piperazine ring. Both the nitrogen atoms of the piperazine are substituted by the P(O)(OC_6_H_5_)_2_ phosphoester moieties (Fig. 1). The phosphorus atom has a distorted tetrahedral configuration with the bond angles in the range of 99.04 (4)° [O(2)–P(1)–O(3)] to 115.93 (5)° [O(1)–P(1)–O(2)]. As observed in related compounds containing piperazine ring substituted by phosphorus (Chen *et al.*, 2007; Balakrishna *et al.*, 2003, 2006; Rodriguez i Zubiri *et al.*, 2002), the N atom shows some deviation from planarity, indeed it is 0.25 (1)Å above the C1, C2, P1 plane.

The oxygen atom of P═O group are involved in a C–H···O hydrogen bond building a R_2_^2^(22) ring (Etter *et al.*, 1990; Bernstein *et al.*, 1995) (Table 1, Fig. 2). Furthermore, these rings are interconnected building infinite chains parallel to the *a* axis. C–H···π interactions involving the phenyl rings stabilize the packing (Table 1).

### Experimental

To a solution of (C_6_H_5_O)_2_P(O)Cl in chloroform, a solution of piperazine and triethylamine (2:1:2 mole ratio) in chloroform was added at 273 K. After 4 h stirring, the solvent was removed and product was washed with distilled water and recrystallized from chloroform/n-heptane at room temperature. IR (KBr, cm^-1^): 3051.2, 2913.8, 2866.3, 1592.5, 1490.4, 1383.3, 1334.6, 1261.7, 1198.5, 1135.3, 1067.1, 1013.7, 969.8, 931.6, 776.8, 686.8. Raman (cm^-1^): 3066.5, 2526.4, 1593.0, 1469.5, 1452.2, 1265.1, 1218.8, 1168.7, 1157.1, 1024.0, 1006.6, 939.2, 771.4, 705.8, 617.1, 414.6, 262.2. ^31^P{^1^H} NMR (202.45 MHz, DMSO-d6, 300.0 K, H_3_PO_4_ external): -1.45 p.p.m. (*s*). ^1^H NMR (500.13 MHz, DMSO-d6, 300.0 K, TMS): 3.01 (*s*, 8H, CH_2_), 7.15–7.27 (*m*, 12H, Ar—H), 7.38–7.40 p.p.m. (*m*, 8H, Ar—H). ^13^C NMR (125.75 MHz, DMSO-d6, 300.0 K, TMS): 43.98 (*d*, ^2(&3)^J(P,C) = 3.5 Hz, 4 C), 119.94 (*d*, ^3^J(P,C) = 4.7 Hz, 8 C, C*_ortho_*), 125.11 (*s*), 129.90 (*s*), 150.07 p.p.m. (*d*, ^2^J(P,C) = 6.4 Hz, 4 C, C*_ipso_*).

### Refinement

H atoms were placed in calculated positions and included in the refinement in a riding-model approximation with C–H = 0.93–0.97 Å, and *U*_iso_(H) = 1.2Ueq(C).

### Crystal data

**Table:**

C_28_H_28_N_2_O_6_P_2_	*F*(000) = 576
*M**_r_* = 550.46	*D*_x_ = 1.425 Mg m^−^^3^
Monoclinic, *P*2_1_/*n*	Mo *K*α radiation, λ = 0.71073 Å
Hall symbol: -P 2yn	Cell parameters from 2258 reflections
*a* = 6.3117 (4) Å	θ = 3–29°
*b* = 8.9530 (5) Å	µ = 0.22 mm^−^^1^
*c* = 22.8630 (13) Å	*T* = 100 K
β = 96.756 (1)°	Plate, colourless
*V* = 1282.99 (13) Å^3^	0.55 × 0.40 × 0.20 mm
*Z* = 2	

### Data collection

**Table:**

Bruker APEXII CCD area-detector diffractometer	3405 independent reflections
Radiation source: fine-focus sealed tube	3071 reflections with *I* > 2σ(*I*)
graphite	*R*_int_ = 0.025
ω scans	θ_max_ = 29.0°, θ_min_ = 1.8°
Absorption correction: multi-scan (*SADABS*; Bruker, 2005)	*h* = −8→8
*T*_min_ = 0.890, *T*_max_ = 0.958	*k* = −12→12
14864 measured reflections	*l* = −30→31

### Refinement

**Table:**

Refinement on *F*^2^	Primary atom site location: structure-invariant direct methods
Least-squares matrix: full	Secondary atom site location: difference Fourier map
*R*[*F*^2^ > 2σ(*F*^2^)] = 0.032	Hydrogen site location: inferred from neighbouring sites
*wR*(*F*^2^) = 0.089	H-atom parameters constrained
*S* = 1.03	*w* = 1/[σ^2^(*F*_o_^2^) + (0.0496*P*)^2^ + 0.567*P*] where *P* = (*F*_o_^2^ + 2*F*_c_^2^)/3
3405 reflections	(Δ/σ)_max_ = 0.002
172 parameters	Δρ_max_ = 0.51 e Å^−^^3^
0 restraints	Δρ_min_ = −0.27 e Å^−^^3^

### Special details

**Table:**

Geometry. All e.s.d.'s (except the e.s.d. in the dihedral angle between two l.s. planes) are estimated using the full covariance matrix. The cell e.s.d.'s are taken into account individually in the estimation of e.s.d.'s in distances, angles and torsion angles; correlations between e.s.d.'s in cell parameters are only used when they are defined by crystal symmetry. An approximate (isotropic) treatment of cell e.s.d.'s is used for estimating e.s.d.'s involving l.s. planes.
Refinement. Refinement of *F*^2^ against ALL reflections. The weighted *R*-factor *wR* and goodness of fit *S* are based on *F*^2^, conventional *R*-factors *R* are based on *F*, with *F* set to zero for negative *F*^2^. The threshold expression of *F*^2^ > σ(*F*^2^) is used only for calculating *R*-factors(gt) *etc*. and is not relevant to the choice of reflections for refinement. *R*-factors based on *F*^2^ are statistically about twice as large as those based on *F*, and *R*- factors based on ALL data will be even larger.

### Fractional atomic coordinates and isotropic or equivalent isotropic displacement parameters (Å^2^)

**Table:**

	*x*	*y*	*z*	*U*_iso_*/*U*_eq_	
P1	0.29408 (4)	0.70141 (3)	0.584588 (12)	0.01226 (9)	
O1	0.08499 (13)	0.65211 (10)	0.59873 (4)	0.01722 (18)	
O2	0.30060 (14)	0.86102 (9)	0.55434 (4)	0.01595 (17)	
O3	0.46851 (13)	0.72855 (9)	0.63989 (3)	0.01431 (17)	
N1	0.40456 (15)	0.59125 (11)	0.54033 (4)	0.01435 (19)	
C1	0.27230 (18)	0.49056 (13)	0.50046 (5)	0.0154 (2)	
H1A	0.2168	0.5452	0.4642	0.019*	
H1B	0.1490	0.4557	0.5198	0.019*	
C2	0.59794 (18)	0.64262 (13)	0.51554 (5)	0.0159 (2)	
H2A	0.6852	0.7054	0.5448	0.019*	
H2B	0.5564	0.7039	0.4800	0.019*	
C3	0.24646 (19)	0.99346 (12)	0.58193 (5)	0.0141 (2)	
C4	0.03688 (19)	1.04226 (13)	0.57405 (5)	0.0174 (2)	
H4A	−0.0717	0.9831	0.5531	0.021*	
C5	−0.0111 (2)	1.18012 (14)	0.59758 (6)	0.0213 (2)	
H5A	−0.1538	1.2157	0.5925	0.026*	
C6	0.1478 (2)	1.26566 (14)	0.62831 (6)	0.0224 (3)	
H6A	0.1142	1.3603	0.6436	0.027*	
C7	0.3561 (2)	1.21302 (14)	0.63680 (6)	0.0221 (3)	
H7A	0.4642	1.2709	0.6586	0.026*	
C8	0.40715 (19)	1.07579 (14)	0.61346 (5)	0.0181 (2)	
H8A	0.5494	1.0393	0.6191	0.022*	
C9	0.51165 (18)	0.61794 (12)	0.68335 (5)	0.0136 (2)	
C10	0.36396 (19)	0.58904 (13)	0.72215 (5)	0.0160 (2)	
H10A	0.2295	0.6380	0.7181	0.019*	
C11	0.4174 (2)	0.48638 (14)	0.76736 (5)	0.0190 (2)	
H11A	0.3176	0.4638	0.7941	0.023*	
C12	0.6154 (2)	0.41695 (14)	0.77356 (5)	0.0203 (2)	
H12A	0.6518	0.3486	0.8049	0.024*	
C13	0.7607 (2)	0.44750 (14)	0.73384 (6)	0.0204 (2)	
H13A	0.8957	0.3993	0.7380	0.025*	
C14	0.70950 (18)	0.54823 (13)	0.68798 (5)	0.0168 (2)	
H14A	0.8075	0.5688	0.6605	0.020*	

### Atomic displacement parameters (Å^2^)

**Table:**

	*U*^11^	*U*^22^	*U*^33^	*U*^12^	*U*^13^	*U*^23^
P1	0.01325 (14)	0.01125 (14)	0.01239 (14)	0.00080 (10)	0.00199 (10)	−0.00088 (9)
O1	0.0147 (4)	0.0193 (4)	0.0182 (4)	0.0001 (3)	0.0038 (3)	−0.0014 (3)
O2	0.0226 (4)	0.0125 (4)	0.0133 (4)	0.0031 (3)	0.0041 (3)	−0.0003 (3)
O3	0.0169 (4)	0.0121 (4)	0.0136 (4)	−0.0012 (3)	−0.0001 (3)	0.0006 (3)
N1	0.0137 (4)	0.0127 (4)	0.0170 (5)	−0.0015 (3)	0.0031 (3)	−0.0035 (3)
C1	0.0135 (5)	0.0158 (5)	0.0170 (5)	−0.0008 (4)	0.0017 (4)	−0.0036 (4)
C2	0.0165 (5)	0.0133 (5)	0.0187 (5)	−0.0013 (4)	0.0054 (4)	−0.0029 (4)
C3	0.0207 (5)	0.0103 (5)	0.0116 (5)	0.0014 (4)	0.0039 (4)	0.0008 (4)
C4	0.0195 (5)	0.0157 (5)	0.0166 (5)	0.0005 (4)	0.0005 (4)	0.0003 (4)
C5	0.0238 (6)	0.0170 (6)	0.0240 (6)	0.0060 (5)	0.0065 (5)	0.0036 (5)
C6	0.0344 (7)	0.0118 (5)	0.0229 (6)	0.0006 (5)	0.0112 (5)	−0.0009 (4)
C7	0.0290 (6)	0.0170 (6)	0.0206 (6)	−0.0073 (5)	0.0049 (5)	−0.0026 (4)
C8	0.0191 (5)	0.0177 (6)	0.0179 (5)	−0.0020 (4)	0.0033 (4)	0.0012 (4)
C9	0.0165 (5)	0.0107 (5)	0.0130 (5)	−0.0004 (4)	−0.0004 (4)	−0.0008 (4)
C10	0.0174 (5)	0.0157 (5)	0.0150 (5)	0.0024 (4)	0.0027 (4)	−0.0012 (4)
C11	0.0249 (6)	0.0178 (5)	0.0148 (5)	0.0005 (5)	0.0045 (4)	0.0005 (4)
C12	0.0268 (6)	0.0166 (6)	0.0167 (5)	0.0024 (5)	−0.0011 (5)	0.0019 (4)
C13	0.0181 (5)	0.0180 (6)	0.0245 (6)	0.0037 (4)	−0.0006 (5)	−0.0001 (5)
C14	0.0154 (5)	0.0157 (5)	0.0195 (5)	0.0000 (4)	0.0021 (4)	−0.0016 (4)

### Geometric parameters (Å, °)

**Table:**

P1—O1	1.4633 (9)	C5—C6	1.3855 (19)
P1—O2	1.5901 (9)	C5—H5A	0.9500
P1—O3	1.5944 (8)	C6—C7	1.3885 (19)
P1—N1	1.6275 (10)	C6—H6A	0.9500
O2—C3	1.4040 (13)	C7—C8	1.3923 (17)
O3—C9	1.4066 (13)	C7—H7A	0.9500
N1—C1	1.4702 (14)	C8—H8A	0.9500
N1—C2	1.4783 (14)	C9—C10	1.3847 (16)
C1—C2^i^	1.5155 (16)	C9—C14	1.3889 (16)
C1—H1A	0.9900	C10—C11	1.3946 (16)
C1—H1B	0.9900	C10—H10A	0.9500
C2—C1^i^	1.5155 (16)	C11—C12	1.3880 (18)
C2—H2A	0.9900	C11—H11A	0.9500
C2—H2B	0.9900	C12—C13	1.3913 (18)
C3—C4	1.3846 (16)	C12—H12A	0.9500
C3—C8	1.3855 (17)	C13—C14	1.3919 (17)
C4—C5	1.3937 (17)	C13—H13A	0.9500
C4—H4A	0.9500	C14—H14A	0.9500
			
O1—P1—O2	115.93 (5)	C6—C5—H5A	119.7
O1—P1—O3	115.30 (5)	C4—C5—H5A	119.7
O2—P1—O3	99.04 (4)	C5—C6—C7	120.00 (11)
O1—P1—N1	114.71 (5)	C5—C6—H6A	120.0
O2—P1—N1	103.85 (5)	C7—C6—H6A	120.0
O3—P1—N1	106.20 (5)	C6—C7—C8	120.27 (12)
C3—O2—P1	122.92 (7)	C6—C7—H7A	119.9
C9—O3—P1	120.74 (7)	C8—C7—H7A	119.9
C1—N1—C2	112.78 (9)	C3—C8—C7	118.71 (11)
C1—N1—P1	120.23 (8)	C3—C8—H8A	120.6
C2—N1—P1	118.91 (7)	C7—C8—H8A	120.6
N1—C1—C2^i^	110.39 (9)	C10—C9—C14	122.29 (11)
N1—C1—H1A	109.6	C10—C9—O3	119.68 (10)
C2^i^—C1—H1A	109.6	C14—C9—O3	117.89 (10)
N1—C1—H1B	109.6	C9—C10—C11	118.39 (11)
C2^i^—C1—H1B	109.6	C9—C10—H10A	120.8
H1A—C1—H1B	108.1	C11—C10—H10A	120.8
N1—C2—C1^i^	109.99 (9)	C12—C11—C10	120.49 (11)
N1—C2—H2A	109.7	C12—C11—H11A	119.8
C1^i^—C2—H2A	109.7	C10—C11—H11A	119.8
N1—C2—H2B	109.7	C11—C12—C13	119.99 (11)
C1^i^—C2—H2B	109.7	C11—C12—H12A	120.0
H2A—C2—H2B	108.2	C13—C12—H12A	120.0
C4—C3—C8	121.97 (11)	C12—C13—C14	120.44 (11)
C4—C3—O2	119.17 (10)	C12—C13—H13A	119.8
C8—C3—O2	118.76 (10)	C14—C13—H13A	119.8
C3—C4—C5	118.47 (11)	C9—C14—C13	118.39 (11)
C3—C4—H4A	120.8	C9—C14—H14A	120.8
C5—C4—H4A	120.8	C13—C14—H14A	120.8
C6—C5—C4	120.55 (12)		

Symmetry codes: (i) −*x*+1, −*y*+1, −*z*+1.

### Hydrogen-bond geometry (Å, °)

**Table:**

Cg1 and Cg2 are the centroids of the C3–C8 and C11–C14 rings, respectively.

**Table:**

*D*—H···*A*	*D*—H	H···*A*	*D*···*A*	*D*—H···*A*
C14—H14A···O1^ii^	0.95	2.49	3.4327 (15)	172
C11—H11A···Cg1^iii^	0.95	2.74	3.3324 (12)	121
C7—H7A···Cg2^iv^	0.95	2.59	3.4099 (13)	145

Symmetry codes: (ii) *x*+1, *y*, *z*; (iii) −*x*+1/2, *y*−1/2, −*z*+3/2; (iv) *x*, *y*+1, *z*.

## References

[bb1] Balakrishna, M. S., George, P. P. & Mague, J. T. (2003). *J. Chem. Res.* pp. 576–577.

[bb2] Balakrishna, M. S., George, P. P. & Mague, J. T. (2006). *Phosphorus Sulfur Silicon Relat. Elem.* **181**, 141–146.

[bb3] Bernstein, J., Davis, R. E., Shimoni, L. & Chang, N.-L. (1995). *Angew. Chem. Int. Ed. Engl.* **34**, 1555–1573.

[bb4] Bruker (2005). *APEX2*, *SAINT* and *SADABS* Bruker AXS Inc., Madison, Wisconsin, USA.

[bb5] Burnett, M. N. & Johnson, C. K. (1996). *ORTEPIII* Report ORNL-6895. Oak Ridge National Laboratory, Tennessee, USA.

[bb6] Chen, X., Xiao, W. & Jiao, P. (2007). *Acta Cryst.* E**63**, o4271.

[bb7] Etter, M. C., MacDonald, J. C. & Bernstein, J. (1990). *Acta Cryst.* B**46**, 256–262. 10.1107/s0108768189012929 2344397

[bb8] Farrugia, L. J. (1997). *J. Appl. Cryst.* **30**, 565.

[bb9] Nguyen, C. & Kim, J. (2008). *Polym. Degrad. Stabil.* **93**, 1037–1043.

[bb10] Rodriguez i Zubiri, M., Slawin, A. M. Z., Wainwright, M. & Woollins, J. D. (2002). *Polyhedron*, **21**, 1729–1736.

[bb11] Sheldrick, G. M. (2008). *Acta Cryst.* A**64**, 112–122. 10.1107/S0108767307043930 18156677

[bb12] Spek, A. L. (2009). *Acta Cryst.* D**65**, 148–155. 10.1107/S090744490804362X PMC2631630 19171970

